# CLUE: A Fast Parallel Clustering Algorithm for High Granularity Calorimeters in High-Energy Physics

**DOI:** 10.3389/fdata.2020.591315

**Published:** 2020-11-27

**Authors:** Marco Rovere, Ziheng Chen, Antonio Di Pilato, Felice Pantaleo, Chris Seez

**Affiliations:** ^1^ European Organization for Nuclear Research (CERN), Meyrin, Switzerland; ^2^ Northwestern University, Evanston, IL, United States; ^3^ University of Bari, Bari, Italy; ^4^ National Institute for Nuclear Physics (INFN)—Sezione di Bari, Bari, Italy; ^5^ Imperial College London, South Kensington Campus, London, United Kingdom

**Keywords:** graphics processing unit, clustering, density, calorimeters, high granularity, HL-LHC, FCC

## Abstract

One of the challenges of high granularity calorimeters, such as that to be built to cover the endcap region in the CMS Phase-2 Upgrade for HL-LHC, is that the large number of channels causes a surge in the computing load when clustering numerous digitized energy deposits (hits) in the reconstruction stage. In this article, we propose a fast and fully parallelizable density-based clustering algorithm, optimized for high-occupancy scenarios, where the number of clusters is much larger than the average number of hits in a cluster. The algorithm uses a grid spatial index for fast querying of neighbors and its timing scales linearly with the number of hits within the range considered. We also show a comparison of the performance on CPU and GPU implementations, demonstrating the power of algorithmic parallelization in the coming era of heterogeneous computing in high-energy physics.

## 1. Introduction

Calorimeters with high lateral and longitudinal readout granularity, capable of providing a fine grained image of electromagnetic and hadronic showers, have been suggested for future high-energy physics experiments (CALICE Collaboration, 2012). The silicon sensor readout cells of the CMS endcap calorimeter (HGCAL) (CMS Collaboration, 2017) for HL-LHC (Apollinari et al., 2017) have an area of about $1\;{\text{cm}}^{2}$. When a particle showers, the deposited energy is collected by the sensors on the layers that the shower traverses. The purpose of the clustering algorithm when applied to shower reconstruction is to group together individual energy deposits (hits) originating from a particle shower. Due to the high lateral granularity, the number of hits per layer is large, and it is computationally advantageous to collect together hits in 2D clusters layer-by-layer (Chen et al., 2017) and then associate these 2D clusters in different layers (CMS Collaboration, 2017).

However, a computational challenge emerges as a consequence of the large data scale and limited time budget. Event reconstruction is tightly constrained by a millisecond-level execution time. This constraint requires the clustering algorithm to be highly efficient while maintaining a low computational complexity. Furthermore, a linear scalability is strongly desired in order to avoid bottlenecking the performance of the entire event reconstruction. Finally, it is highly preferable to have a fully parallelizable clustering algorithm to take advantage of the trend of heterogeneous computing with hardware accelerators, such as graphics processing units (GPUs), achieving a higher event throughput and a better energy efficiency.

The input to the clustering algorithm is a set of *n* hits, whose number varies from a few thousands to a few millions, depending on the longitudinal and transverse granularity of the calorimeter as well as on the number of particles entering the detector. The output is a set of *k* clusters whose number is usually one or two orders of magnitude smaller than *n* and in principle depends on both the number of incoming particles and the number of layers. Assuming that the lateral granularity of sensors is constant and finite, the average number of hits in clusters (m=n/k) is also constant and finite. For example, in the CMS HGCAL, *m* is in the order of 10. This leads to the relation among the number of hits *n*, the number of clusters *k*, and the average number of hits in clusters *m* as n>k≫m.

Most well-known algorithms do not simultaneously satisfy the requirements on linear scalability and easy parallelization for applications such as clustering hits in high granularity calorimeters, which is characterized by low dimension and n>k≫m. It is therefore important to investigate new, fast, and parallelizable clustering algorithms, as well as their optimized accompanying spatial index that can be conveniently constructed and queried in parallel.

In this study, we describe CLUstering of Energy (CLUE), a novel and parallel density-based clustering. Its development was inspired by the work described in ref. (Rodriguez and Laio, 2014). In Section 2, we describe the CLUE algorithm and its accompanying spatial index. Then in Section 3, some details of GPU implementations are discussed. Finally, in Section 4 we present CLUE’s ability on nonspherical cluster shapes and noise rejection, followed by its computational performance when executed on CPU and GPU with synthetic data, mimicking hits in high granularity calorimeters.

## 2. Clustering Algorithm

Clustering data is one of the most challenging tasks in several scientific domains. The definition of cluster is itself not trivial, as it strongly depends on the context. Many clustering methods have been developed based on a variety of induction principles (Maimon and Rokach, 2005). Currently popular clustering algorithms include (but are not limited to) partitioning, hierarchical, and density-based approaches (Maimon and Rokach, 2005; Han et al., 2012). Partitioning approaches, such as k-mean (Lloyd, 1982), compose clusters by optimizing a dissimilarity function based on distance. However, in the application to high granularity calorimeters, partitioning approaches are prohibitive because the number of clusters *k* is not known a priori. Hierarchical methods make clusters by constructing a dendrogram with a recursion of splitting or merging. However, hierarchical methods do not scale well because each decision to merge or split needs to scan over many objects or clusters (Han et al., 2012). Therefore, they are not suitable for our application. Density-based methods, such as DBSCAN (Ester et al., 1996), OPTICS (Ankerst et al., 1999), and Clustering by Fast Search and Find Density Peak (CFSFDP) (Rodriguez and Laio, 2014), group points by detecting continuous high-density regions. They are capable of discovering clusters of arbitrary shapes and are efficient for large spatial database. If a spatial index is used, their computational complexity is O(nlogn) (Han et al., 2012). However, one of the potential weaknesses of the currently well-known density-based algorithms is that they intrinsically include serial processes that are hard to parallelize: DBSCAN has to iteratively visit all points within an enclosure of density-connectedness before working on the next cluster (Ester et al., 1996); OPTICS needs to sequentially add points in an ordered list to obtain a dendrogram of reachability distance (Ankerst et al., 1999); CFSFDP needs to sequentially assign points to clusters in order of decreasing density (Rodriguez and Laio, 2014). In the application to high granularity calorimeters, as discussed in Section 1, linear scalability and full parallelization are essential to handle a huge dataset efficiently by means of heterogeneous computing.

In order to satisfy these requirements, we propose a fast and fully parallelizable density-based algorithm (CLUE) inspired by CFSFDP. For the purpose of the algorithm, each sensor cell on a layer with its energy deposit is taken as a 2D point with an associated weight equaling its energy value. As in CFSFDP, two key variables are calculated for each point: the local density *ρ* and the separation *δ* defined in Eqs 3 **and** 4, where *δ* is the distance to the nearest point with higher density (“nearest-higher”), which is slightly adapted from that in CFSFDP in order to take advantage of the spatial index. Then cluster seeds and outliers are identified based on thresholds on *ρ* and *δ*. Differing from cluster assignment in CFSFDP, which sorts density and adds points to clusters in order of decreasing density, CLUE first builds a list of followers for each point by registering each point as a follower to its nearest-higher. Then it expands clusters by passing cluster indices from the seeds to their followers iteratively. Since such expansion of clusters is fully independent from each others', it not only avoids the costly density sorting in CFSFDP, but also enables a *k*-way parallelization. Unlike the noise identification in CFSFDP, CLUE rejects noise by identifying outliers and their iteratively descendant followers, as discussed in Section 4.1.

### 2.1. Spatial Index With Fixed-Grid

Query of neighborhood, which retrieves nearby points within a distance, is one of the most frequent operations in density-based clustering algorithms. CLUE uses a spatial index to access and query spatial data points efficiently. Given that the physical layout of sensor cells is a multi-layer tessellation, it is intuitive to index its data with a fixed-grid, which divides the space into fixed rectangular bins (Levinthal, 1966; Bentley and Friedman, 1979). Comparing with the data-driven structures such as KD-Tree (Bentley, 1975) and R-Tree (Guttman, 1984), space partition in fixed-grid is independent of any particular distribution of data points (Rigaux et al., 2001), thus can be explicitly predefined before loading data points. In addition, both construction and query with a fixed-grid are computationally simple and can be easily parallelized. Therefore, CLUE uses a fixed-grid as spatial index for efficient neighborhood queries.

**FIGURE 1:**
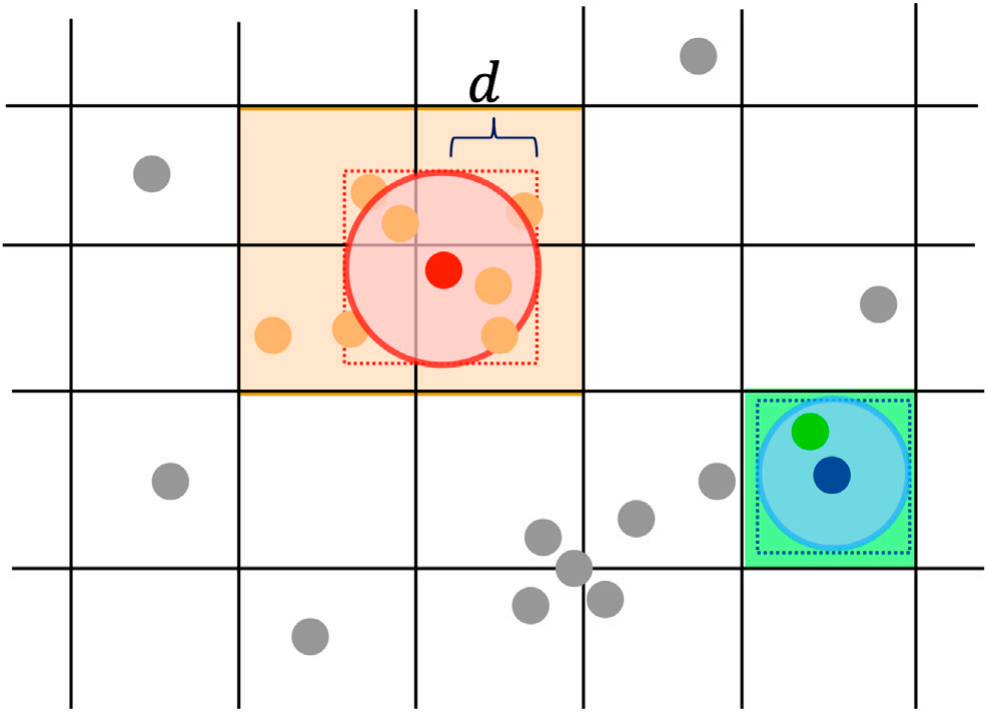
2D points are indexed with a grid for fast neighborhood query in CLUE. Construction of this spatial index only involves registering the indices of points into the bins of the grid according to points’ 2D spatial positions. To query d-neighborhood $N_{d}\left(i\right)$ defined in Eq. 2, taking the red (blue) point for example, we first locate its ${\text{Ω}}_{d}\left(i\right)$ defined in Eq. 1, a set of all points in the bins touched by a square window $\left[x_{i}\pm d,\;y_{i}\pm d\right]$. The $\left[x_{i}\pm d,y_{i}\pm d\right]$ window is shown as the orange (green) square, while ${\text{Ω}}_{d}\left(i\right)$ is shown as orange (green) points. Then, we examine points in ${\text{Ω}}_{d}\left(i\right)$ to identify those within a distance *d* from point *i*, shown as the ones contained in the red (blue) circle.

For each layer of the calorimeter, a fixed-grid spatial index is constructed by registering the indices of 2D points into the square bins in the grid according to the 2D coordinates of the points. When querying $N_{d}\left(i\right)$, the d-neighborhood of point *i*, CLUE only needs to loop over points in the bins touched by the square window $\left(x_{i}\pm d,y_{i}\pm d\right)$ as shown in Figure 1. We denote those points as ${\text{Ω}}_{d}\left(i\right)$, defined as:

$${\text{Ω}}_{d}\left(i\right)=\left\{j:j\in \text{tiles touched by the square window }\left[x_{i}\pm d,y_{i}\pm d\right]\right\}.$$ (1)

Here, ${\text{Ω}}_{d}\left(i\right)$ is guaranteed to include all neighbors within a distance *d* from the point *i*. Namely,

$$N_{d}\left(i\right)=\left\{j:d_{ij}<d,j\in {\text{Ω}}_{d}\left(i\right)\right\}\subseteq {\text{Ω}}_{d}\left(i\right).$$ (2)

Here, $d_{ij}$ is the distance between points *i* and *j*. Without any spatial index, the query of $N_{d}\left(i\right)$ requires a sequential scan over all points. In contrast, with the grid spatial index, CLUE only needs to loop over the points in ${\text{Ω}}_{d}\left(i\right)$ to acquire $N_{d}\left(i\right)$. Given that *d* is small and the maximum granularity of points is constant, the complexity of querying $N_{d}\left(i\right)$ with a fixed-grid is O(1).

### 2.2. Clustering Procedure of CLUE

CLUE requires the following four parameters: $d_{c}$ is the cut-off distance in the calculation of local density; $\rho_{c}$ is the minimum density to promote a point as a seed or the maximum density to demote a point as an outlier; $\delta_{c}$ and $\delta_{o}$ are the minimum separation requirements for seeds and outliers, respectively. The choice of these four parameters can be based on physics: for example, $d_{c}$ can be chosen based on the shower size and the lateral granularity of detectors; $\rho_{c}$ can be chosen to exclude noise; $\delta_{c}$ and $\delta_{o}$ can be chosen based on the shower sizes and separations. These four parameters allow more degrees of freedom to tune CLUE for the desired goals of physics.

**FIGURE 2:**
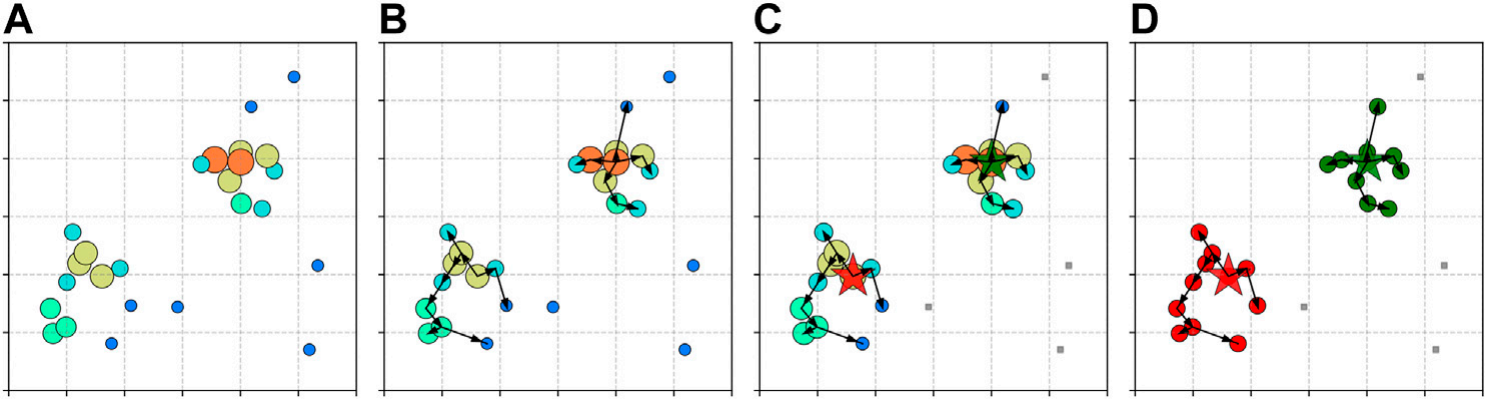
Demonstration of CLUE algorithm. Points are distributed inside a 6×6 2D area and CLUE parameters are set to $d_{c}=0.5,\;\rho_{c}=3.9,\;\delta_{c}=\delta_{o}=1$. Before the clustering procedure starts, a fixed-grid spatial index is constructed. In the first step, shown as **(A)**, CLUE calculates the local density *ρ* for each point, which is defined in Eq. 3. The color and size of points represent their local densities. In the second step, shown as **(B)**, CLUE calculates the nearest-higher nh and the separation *δ* for each point, which are defined in Eq. 4. The black arrows represent the relation from the nearest-higher of a point to the point itself. If the nearest-higher of a point is −1, there is no arrow pointing to it. In the third step, shown as **(C)**, CLUE promotes a point as a seed if ρ, δ are both large, or demote it to an outlier if *ρ* is small and *δ* is large. Promoted seeds and demoted outliers are shown as stars and gray squares, respectively. In the fourth step, shown as **(D)**, CLUE propagates the cluster indices from seeds through their chains of followers defined in Eq. 5. Noise points, which are outliers and their descendant followers, are guaranteed not to receive any cluster ids from any seeds. The color of points represents the cluster ids. A gray square means its cluster id is undefined and the point should be considered as noise.

Figure 2 illustrates the main steps of CLUE algorithm. The local density *ρ* in CLUE is defined as:

$$\rho_{i}=\underset{j:j\in N_{d_{c}}\left(i\right)}{\sum }\chi \left(d_{ij}\right)w_{j},$$ (3)

where $w_{j}$ is the weight of point *j*, $\chi \left(d_{ij}\right)$ is a convolution kernel, which can be optimized according to specific applications. Obvious possible kernel options include flat, Gaussian, and exponential functions.

The nearest-higher and the distance to it *δ* (separation) in CLUE are defined as:

$$nh_{i}=\{\begin{array}{ll} \text{arg}\underset{j\in N_{d_{m}}^{\prime }\left(i\right)}{\text{min}}d_{ij}, & \text{if }|N\prime_{d_{m}}\left(i\right)|\neq 0\\ -1, & \text{otherwise} \end{array},\;\delta_{i}=\{\begin{array}{ll} d_{i,nh_{i}}, & \text{if }|N\prime_{d_{m}}\left(i\right)|\neq 0\\ +\infty , & \text{otherwise} \end{array},$$ (4)

where $d_{m}=\text{max}\left(\delta_{o},\delta_{c}\right)$ and $N\prime_{d_{m}}\left(i\right)=\left\{j:\rho_{j}>\rho_{i},j\in N_{d_{m}}\left(i\right)\right\}$ is a subset of $N_{d_{m}}\left(i\right)$, where points have higher local densities than $\rho_{i}$.

After *ρ* and *δ* are calculated, points with density $\rho >\rho_{c}$ and large separation $\delta >\delta_{c}$ are promoted as cluster seeds, while points with density $\rho <\rho_{c}$ and large separation $\delta >\delta_{o}$ are demoted to outliers. For each point, there is a list of followers defined as:

$$F_{i}=\left\{j:nh_{j}=i\right\}.$$ (5)

**FIGURE 3:**
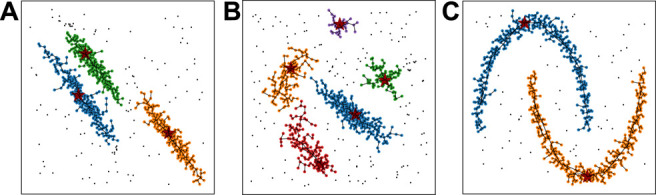
Examples of CLUE clustering on synthetic datasets. Each sample includes 1000 2D points with the same weight generated from certain distributions, including uniform noise points. The color of points represent their cluster ids. Black points represent outliers detached from any clusters. The links between pairs of points illustrate the relationship between nearest-higher and follower. The red stars highlight the cluster seeds.

The lists of followers are built by registering the points that are neither seeds nor outliers to the follower lists of their nearest-highers. The cluster indices, associating a follower with a particular seed, are passed down from seeds through their chains of followers iteratively. Outliers and their descendant followers are guaranteed not to receive any cluster indices from seeds, which grants a noise rejection as shown in Figure 3. The calculation of ρ,δ, and the decision of seeds and outliers both support *n*-way parallelization, while the expansion of clusters can be done with *k*-way parallelization. Pseudocode of CLUE is included in Supplementary Material.

## 3. GPU Implementation

To parallelize CLUE on GPU, one GPU thread is assigned to each point, for a total of *n* threads, to construct spatial index, calculate *ρ* and *δ*, promote (demote) seeds (outliers), and register points to the corresponding lists of followers of their nearest-highers. Next, one thread is assigned to each seed, for a total of *k* threads, to expand clusters iteratively along chains of followers. The block size of all kernels, which in practice does not have a remarkable impact on the speed performance, is set to 1,024. In the test in Table 1, changing the block size from 1,024 to 256 on GPU leads to only about 0.14 ms decrease in the sum of kernel execution times. The details of parallelism for each kernel are listed in Table 2. Since the results of a CLUE step are required in the following steps, it is necessary to guarantee that all the threads are synchronized before moving to the next stage. Therefore, each CLUE step can be implemented as a separate kernel. To optimize the performance of accessing the GPU global memory with coalescing, the points on all layers are stored as a single structure-of-array (SoA), including information of their layer numbers and 2D coordinates and weights. Thus, points on all layers are input into kernels in one shot. The total memory required to run CLUE with up to 1 M hits is about 284 MB. This includes the memory needed to store the input data, the output results, and all the intermediate structures needed by the algorithm.

**TABLE 1:** Decomposition of CLUE execution time in the case of $10^{4}$ points per layer with 100 layers. The time of subprocesses on GPU is measured with NVIDIA profiler, while that on CPU is measured with std::chrono timers in the C++ code. The uncertainties are the standard deviations of 200 trial runs of the same event (10,000 trial runs if GPU). The uncertainties of subprocesses on GPU are negligible given that the maximum and minimum kernel execution time measured by NVIDIA Profiler are very close. With respect to the single-threaded CPU, the speed-up factors of the multi-threaded CPU with TBB and the GPU are given in the bracket. “mem mgmt + overhead” represents the time spent in handling and copying data, together with the overhead of issuing instructions to the GPU.

CLUE step	CPU [1T] (baseline)	CPU TBB [10T]	GPU
Build fixed-grid spatial index	59.3 ± 1.6 ms	117.7 ± 6.4 ms (0.50x)	0.28 ms (208.6x)
Calculate local density	218.4 ± 2.5 ms	33.7 ± 2.6 ms (6.48x)	0.51 ms (430.6x)
Calculate nearest-higher and separation	326.9 ± 2.9 ms	45.5 ± 2.5 ms (7.19x)	0.89 ms (368.5x)
Decide seeds/outliers, register followers	54.4 ± 2.5 ms	109.4 ± 7.7 ms (0.50x)	0.34 ms (162.4x)
Expand clusters	17.4 ± 1.5 ms	6.1 ± 1.3 ms (2.86x)	0.35 ms (49.7x)
Mem mgmt + overhead	29.1 ± 1.7 ms	44.9 ± 15.7 ms	4.27 ms
TOTAL (10,000 points per layer)	705.5 ± 7.9 ms	357.2 ± 19.7 ms (2.0x)	6.63 ± 0.63 ms (106.4x)

**TABLE 2:** Kernels and parallelism.

Kernels	Parallelism	Total threads	Block size
Build fixed-grid spatial index	1 point/thread	n	1,024
Calculate local density	1 point/thread	n	1,024
Calculate nearest-higher and separation	1 point/thread	n	1,024
Decide seeds/outliers, register followers	1 point/thread	n	1,024
Expand clusters	1 seed/thread	k	1,024

When parallelizing CLUE on GPU, thread conflicts to access and modify the same memory address in global memory could happen in the following three cases:

- multiple points need to register to the same bin simultaneously;
- multiple points need to register to the list of seeds simultaneously;
- multiple points need to register as followers to the same point simultaneously.

Therefore, atomic operations are necessary to avoid the race conditions among threads in the global memory. During an atomic operation, a thread is granted with an exclusive access to read from and write to a memory location that is inaccessible to other concurrent threads until the atomic operation finishes.

This inevitably leads to some microscopic serialization among threads in race. The serialization in cases (i) and (iii) is negligible because bins are usually small as well as the number of followers of a given point. In contrast, serialization in case (ii) can be costly because the number of seeds *k* is large. This can cause delays in the execution of kernel responsible for seed promotion. Since the atomic pushing back to the list of seeds is relatively fast in GPU memory comparing to the data transportation between host and device, the total execution time of CLUE still does not suffer significantly from the serialization in case (ii). The speed performance is further discussed in Section 4.

## 4. Performance Evaluation

### 4.1. Clustering Results



We demonstrate the clustering results of CLUE with a set of synthetic datasets, shown in Figure 3. Each example has 1,000 2D points and includes spatially uniform noise points. The datasets in Figures 3A,C are from the scikit-learn package (Pedregosa et al., 2011). The dataset in Figure 3B is taken from (Rodriguez and Laio, 2014). Figures 3A,B include elliptical clusters and Figure 3C contains two parabolic arcs. CLUE successfully detects density peaks in Figures 3A–C.

**FIGURE 4:**
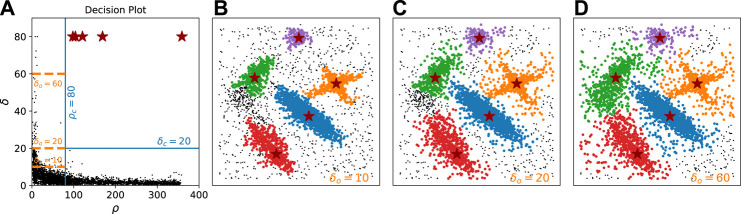
Noise rejection using different values of δo. Noise is either an outlier or a descendant follower of an outlier. In this dataset (Rodriguez & Laio, 2014), 4,000 Points are distributed in 500 × 500 2D square area. **(A)** represents the decision plot on the ρ − δ plane, where fixed ρ_c_ 80 and δ_c_ 40 values are shown as vertical and horizontal blue lines, respectively. Three different values of δ_o_ (10, 20, 60) are shown as orange dash lines. **(B−D)** show the results with δo 10, 20, 60, respectively, illustrating how increasing δo loosens the continuity requirement and helps to demote outliers. The level of denoise should be chosen according to the user's needs.

In the induction principle of density-based clustering, the confidence of assigning a low-density point to a cluster is established by maintaining the continuity of the cluster. Low-density points with large separation should be deprived of association to any clusters. CFSFDP uses a rather costly technique, which calculates a border region of each cluster and defines core-halo points in each cluster, to detach unreliable assignments from clusters (Rodriguez and Laio, 2014). In contrast, CLUE achieves this using cuts on $\delta_{o}$ and $\rho_{c}$, while expanding a cluster, as described in Section 2. The example in Figure 4 shows how cutting at different separation values helps to demote outliers. Figure 4A represents the decision plot on the ρ−δ plane. Points with density below $\rho_{c}=80$, shown on the left side of the vertical blue line, could be demoted as outliers if their δ is larger than a threshold. Once an outlier is demoted, all its descendant followers are disallowed from attaching to any clusters. While keeping $\rho_{c}=80$ fixed, the effect of using three different values of $\delta_{o}$ (10, 20, 60), shown as orange dash lines in Figure 4A, has been investigated. The corresponding results are shown in Figures 4B–D, respectively.

The physics requirements of the clustering for the CMS HGCAL can be summarized as collecting a high fraction of the energy deposited by a single shower in a single cluster. The algorithm should form separate clusters of separate showers even when the showers overlap, as far as the granularity of the detector and the shower lateral size allows, but not split the energy deposited by a single shower into more that one cluster. This requirement is most easily definable for electromagnetic showers that have a regular and repeatable form, and slightly less obvious in hadronic showers, which in the fine granularity of the HGCAL frequently have the form of a branching tree of subshowers.

It is found that the CLUE algorithm can be tuned to well satisfy these physics requirements by adjusting its parameters to the cluster characteristics in the calorimeter. In particular, the convolution kernel described in Section 2.2, is approximated to a highly simplified description of the lateral shower shape.

### 4.2. Execution Time and Scaling

**FIGURE 5:**
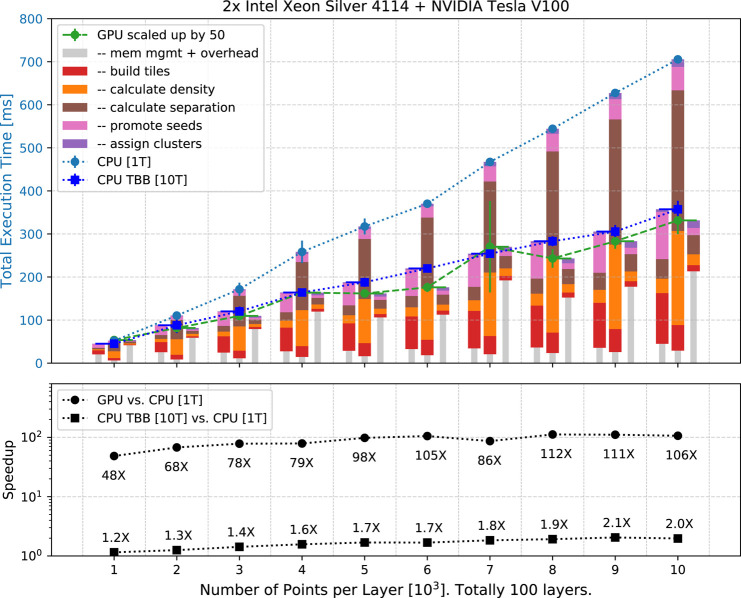
**(Upper)** Execution time of CLUE on the single-threaded CPU, multi-threaded CPU with TBB, and GPU scale linearly with number of input points, ranging from $10^{5}$ to $10^{6}$ in total. Execution time on single-threaded CPU is shown as blue circle dots and on 10 multi-threaded CPU with TBB is shown as blue square dots, while the time on GPU is shown as green circle dots, scaled up by a factor 50 to fit the same vertical scale. The stacked bars represent the decomposition of execution time. The gray narrower bars are latency for data traffic and memory management; wider bars represent time of essential CLUE steps **(Lower)** Comparing with the single-threaded CPU, the speed-up factors of the GPU range from 48 to 112, while the speed-up factors of the multi-threaded CPU with TBB range from 1.2 to 2.0, which is less than the number of concurrent threads on CPU because of atomic pushing to the data containers discussed in Section 3. Table 1 shows the details of the decomposition of the execution time in the case of $10^{4}$ points per layer.

We tested the computational performance of CLUE using a synthetic dataset that resembles high-occupancy events in high granularity calorimeters operated at HL-LHC. The dataset represents a calorimeter with 100 sensor layers. A fixed number of points on each layer are assigned a unit weight in such a way that the density represents circular clusters of energy whose magnitude decreases radially from the center of the cluster according to a Gaussian distribution with the standard deviation, *σ*, set to 3 cm. 5% of the points represent noise distributed uniformly over the layers. When clustering with CLUE, the bin size is set to 5 cm comparable with the width of the clusters, and the algorithm parameters are set to $d_{c}=3\text{ cm},\;\delta_{o}=\delta_{c}=5\text{ cm},\;\rho_{c}=8$. To test CLUE’s linear scaling, the number of points on each layer is incremented from 1,000 to 10,000 in 10 equaling steps. A total of 100 layers are input to CLUE simultaneously, which simulates the proposed CMS HGCAL design (The Phase-2 Upgrade of the CMS Endcap Calorimeter, 2017). Therefore, the total number of points in the test ranges from $10^{5}$ to $10^{6}$. The linear scaling of execution time is validated in Figure 5.

The single-threaded version of the CLUE algorithm on CPU has been implemented in C++, while the one on GPU has been implemented in C with CUDA (Nvidia Corporation, 2010). The multi-threaded version of CLUE on CPU uses the Threading Building Blocks (TBB) library (Reinders, 2007) and has been implemented using the Abstraction Library for Parallel Kernel Acceleration (Alpaka) (Zenker et al., 2016). The test of the execution time is performed on an Intel Xeon Silver 4114 CPU and NVIDIA Tesla V100 GPU connected by PCIe Gen-3 link. The time of each GPU kernel and CUDA API call is measured using the NVIDIA profiler. The total execution time is averaged over 200 identical events (10,000 identical events if GPU). Since CLUE is performed event-by-event and it is not necessary to repeat memory allocation and release for each event when running on GPU, we perform a one-time allocation of enough GPU memory before processing events and a one-time GPU memory deallocation after finishing all events. Therefore, the one-time *cudaMalloc* and *cudaFree* are not included in the average execution time. Such exclusion is legit because the number of events is extremely massive in high-energy physics experiments and the execution time of the one-time *cudaMalloc* and *cudaFree* reused by each individual event is negligible.

In Figure 5 (upper), the scaling of CLUE is linear, consistent with the expectation. The execution time on the single-threaded CPU, multi-threaded CPU with TBB, and GPU increases linearly with the total number of points. The stacked bars represent the decomposition of execution time. In the decomposition, unique to the GPU implementation is the latency of data transfer between host and device, which is accounted for in the grey narrower bar, while common to all the three implementations are the five CLUE steps. Comparing with the single-threaded CPU, when building spatial index and deciding seeds, shown as red and pink bars, the multi-threaded CPU using TBB does not give a notable speed-up due to the implementation of atomic operations in Alpaka (Zenker et al., 2016) as discussed in Section 3, while the GPU has a prominent outperformance thanks to its larger parallelization scale. For the GPU case, the kernel of seed-promotion in which serialization exists due to atomic appending of points in the list of seeds, does not affect the total execution time significantly if compared with other subprocesses. In the two most computing-intense steps, calculating density and separation, there are no thread conflicts or inevitable atomic operations. Therefore, both the multi-threaded CPU using TBB and the GPU provide a significant speed-up. The details of the decomposition of execution time in the case of $10^{4}$ points per layer are listed in Table 1.

Figure 5 (lower) shows the speed-up factors. Compared to the single-threaded CPU, the CUDA implementation on GPU is 48–112 times faster, while the multi-threaded version using TBB via Alpaka with 10 threads on CPU is about 1.2–2.0 times faster. The speed-up factors are constrained to be smaller than the number of concurrent threads because of the atomic operations that introduce serialization. In Table 1, the speed-up factors of multi-threaded CPU using TBB reduce to less than one in the subprocess steps of building spatial index and promoting seeds and registering followers, where atomic operations happen and bottleneck the overall speed-up factor.

## 5. Conclusion

The clustering algorithm is an important part in the shower reconstruction of high granularity calorimeters to identify hot regions of energy deposits. It is required to be computationally linear with data scale *n*, independent from prior knowledge of the number of clusters *k* and conveniently parallelizable when n>k≫m≡n/k in 2D. However, most of the well-known algorithms do not simultaneously support linear scalability and easy parallelization. CLUE is proposed to efficiently perform clustering tasks in low-dimension space with n>k≫m, including (and beyond) the applications in high granularity calorimeters. The clustering time scales linearly with the number of input hits in the range of multiplicity that is relevant for, e.g., the high granularity calorimeter of the CMS experiment at CERN. We evaluated the performance of CLUE on synthetic data and demonstrated its capability on nonspherical cluster shape with adjustable noise rejection. Furthermore, the studies suggest that CLUE on GPU outperforms single-thread CPU by more than an order of magnitude within the data scale ranging from $n=10^{5}$ to $10^{6}$.

## Data Availability

The datasets presented in this study can be found in online repositories. The names of the repository/repositories and accession number(s) can be found below: https://gitlab.cern.ch/kalos/clue/tree/V_01_20.
