# Supplementary material for: CLUE: A Fast Parallel Clustering Algorithm for High Granularity Calorimeters in High-Energy Physics
Source: Front Big Data. 2020 Nov 27;3:591315. doi: 10.3389/fdata.2020.591315 (PMC8080903; doi:10.3389/fdata.2020.591315)
Supplement: Supplementary file 1 [file datasheet1.pdf]

A Pseudocode

Pseudocode of CLUE in serialized implementation.

Algorithm 1: calculate ρ

```
for i ∈ points do
    ρ[i] = 0
    for j ∈ Ω_dc(i) do
        if dist(i, j) < d_c then
            ρ[i] += w[j]
```

Algorithm 2: calculate δ

```
for i ∈ points do
    δ[i] = +∞
    nh[i] = −1
    for j ∈ Ω_dm(i) do
        if dist(i, j) < d_m and ρ[j] > ρ[i] then
            if dist(i, j) < δ[i] then
                nh[i] = j
                δ[i] = d_ij
```

Algorithm 3: find seeds and outliers, assign clusters

```
k = 0;
stack = [] ;
for i ∈ points do
    isSeed = ρ[i] > ρ_c and δ[i] > δ_c
    isOutlier = ρ[i] < ρ_c and δ[i] > δ_o
    if isSeed then
        clusterId[i] = k
        k++
        stack.pushback(i)
    else
        if not isOutlier then
            followers[nh[i]].pushback(i)
while stack.size > 0 do
    i = stack.back
    stack.popback
    for j ∈ followers[i] do
        clusterId[j] = clusterId[i]
        stack.pushback(j)
```
